# Use of antibacterial sutures for skin closure in controlling surgical site infections: a systematic review of published randomized, controlled trials

**DOI:** 10.1093/gastro/got003

**Published:** 2013-03-12

**Authors:** Muhammad S. Sajid, L. Craciunas, P. Sains, K.K. Singh, M.K. Baig

**Affiliations:** Department of General & Laparoscopic Colorectal Surgery, Worthing Hospital, Worthing, West Sussex, BN11 2DH, UK

**Keywords:** wound closure, surgical site infection, antibacterial sutures, operative complications

## Abstract

**Objective:** The objective of this article is to systematically analyse the randomized, controlled trials that compare the use of antibacterial sutures (ABS) for skin closure in controlling surgical site infections.

**Methods:** Randomized, controlled trials on surgical patients comparing the use of ABS for skin closure in controlling the surgical site infections were analysed systematically using RevMan® and combined outcomes were expressed as odds ratios (OR) and standardized mean differences (SMD).

**Results:** Seven randomized, controlled trials evaluating 1631 patients were retrieved from electronic databases. There were 760 patients in the ABS group and 871 patients in the simple suture group. There was moderate heterogeneity among trials (Tau^2^ = 0.12; chi^2^ = 8.40, df = 6 [*P <* 0.01]; I^2^ = 29%). Therefore in the random-effects model, the use of ABS for skin closure in surgical patients was associated with a reduced risk of developing surgical site infections (OR, 0.16; 95% CI, 0.37, 0.99; z = 2.02; *P <* 0.04) and postoperative complications (OR, 0.56; 95% CI, 0.32, 0.98 z = 2.04; *P =* 0.04). The durations of operation and lengths of hospital stay were similar following the use of ABS and SS for skin closure in patients undergoing various surgical procedures.

**Conclusion:** Use of ABS for skin closure in surgical patients is effective in reducing the risk of surgical site infection and postoperative complications. ABS is comparable with SS in terms of length of hospital stay and duration of operation.

## INTRODUCTION

Surgical site infection (SSI) is an immense burden on healthcare resources even in the modern era of immaculate sterilization approaches and highly effective antibiotics. An estimated 234 million various surgical procedures, involving skin incisions requiring various types of wound closure techniques, are performed in the world, with the majority resulting in a wound healing by primary intention [1]. Skin wounds are at risk of SSI and therefore may lead to increased morbidity, delayed recovery and prolonged hospital stay [2]. The prevalence of SSI in the developed world is variable but reported figures are estimated at around 5% [3–4]. The development of SSI is a multifactorial phenomenon, which requires a multimodal approach to prevent and treat it in a timely manner to avoid financial, psychological and health-related quality of life consequences. Various predisposing aetiopathological factors for SSI include immunosupression, nutritional deficiencies, hypoproteinemias, congestive cardiac failure, hepatic failure, renal failure, use of steroids, chemotherapy agents, steroids and diabetes mellitus [5–8]. In additions to these factors, wound contamination, contaminated instruments, surgical technique and sutures used to close skin have also been reported to be responsible for SSI and cosmetic outcomes [9–11]. The prevention of the SSI by various invasive and non-invasive interventions is the most common measure surgeons and other healthcare professional advocate to tackle the problem of SSI. This includes use of prophylactic antibiotics [12–13] and various other multimodal approaches already reported in the medical literature [14–15].

Triclosan [5-chloro-2-(2,4-dichlorophenoxy)phenol] is a broad-spectrum bacteriocidal agent that has been used for more than 40 years in various products, such as toothpaste and soaps. Higher concentrations of triclosan work as a bactericide by attacking different structures in the bacterial cytoplasm and cell membrane [16]. At lower concentrations, triclosan acts as bacteriostatic agent, binding to enoyl-acyl reductase (ENR), a product of the *Fab I* gene and thus inhibiting fatty acid synthesis [17–18]. Use of triclosan-coated sutures should theoretically result in the reduction of SSI. Several studies have shown a reduction in the number of bacteria *in vitro* and also of wound infections in animals [19–21]. The objective of this article is to systematically analyse the randomized, controlled trials comparing the use of triclosan-coated antibacterial sutures (ABS) versus simple sutures (SS) for skin closure in controlling the SSIs. We aimed to include only those trials in which the SSI was investigated as a primary outcome regardless the surgical specialty. The SSI was the primary outcome of this study, whereas postoperative complications, duration of the operation and length of the hospital stay (if reported) were analysed as secondary outcome measures.

## METHODS

### Identification of trials

Randomised, controlled trials (irrespective of language, country of origin, hospital of origin, blinding, sample size or publication status) comparing ABS against SS were included in this review. The Cochrane Colorectal Cancer Group (CCCG) Controlled Trials Register, the Cochrane Central Register of Controlled Trials (CENTRAL) in the Cochrane Library, Medline, Embase and Science Citation Index Expanded were searched for articles published up to October 2012, using the medical subject heading (MeSH) terms “skin closure” and “wound closure” in combination with free text search terms, such as “suture closure”, “sub-cuticular closure”, “absorbable suture”, “non-absorbable suture”, “antibiotic-coated suture”, “triclosan-coated sutures” and “primary wound closure”. A filter for identifying randomized, controlled trials recommended by the Cochrane Collaboration was used to filter out non-randomized studies in Medline and Embase [22]. The references from the included trials were searched to identify additional trials.

### Data extraction

Two authors independently identified the trials for inclusion and exclusion and extracted the data. The accuracy of the extracted data was further confirmed by a third author. There were no discrepancies in the selection of the trials or in data extraction between the reviewers, except in the case of recording the severity of pain according to the measurement scales and timing of the recorded data. All reviewers agreed that blinding was impossible to achieve in the case of the operating surgeon. However, there was disagreement with regard to whether the trials should be classified as having a high or low risk of bias, based on four parameters, namely randomization technique, power calculations, blinding and intention-to-treat analysis. It was agreed that the lack of an adequate randomisation technique and an intention-to-treat analysis would result in the trials being classified as having a high risk of bias. In case of any unclear or missing information, the reviewers planned to obtain those by contacting the authors of the individual trials.

### Statistical analysis

The software package RevMan® 5.1.2 [23], provided by the Cochrane Collaboration, was used for the statistical analysis to achieve a combined outcome. The odds ratio (OR) with a 95% confidence interval (CI) was calculated for binary data and the standardised mean difference (SMD) with a 95% CI was calculated for continuous data variables. The random-effects model was used to calculate the combined outcomes of both binary and continuous variables [24, 25]. Heterogeneity was explored using the chi-squared test, with significance set at *P <* 0.05 and was quantified using I-squared [26], with a maximum value of 30% identifying low heterogeneity [26]. The Mantel-Haenszel method was used for the calculation of RR under the random-effect models [27]. In a sensitivity analysis, 0.5 was added to each cell frequency for trials in which no event occurred in either the treatment or control group, according to the method recommended by Deeks *et al.* [28]. If the standard deviation was not available, it was calculated according to the guidelines of the Cochrane Collaboration [22]. This process involved assumptions that both groups had the same variance—which may not have been true—and variance was either estimated from the range or from the *P*-value. The estimate of the difference between both techniques was pooled, depending upon the effect weights in results determined by each trial estimate variance. A forest plot was used for the graphical display of the results. The square around the estimate stood for the accuracy of the estimation (sample size) and the horizontal line represented the 95% CI. The methodological quality of the included trials was initially assessed using the published guidelines of Jadad *et al.* and Chalmers *et al.* [29–30]. Based on the quality of the included randomized, controlled trials, the strength and summary of the evidence was further evaluated by GradePro® [31], a tool provided by the Cochrane Collaboration.

## RESULTS

**Figure 1:**
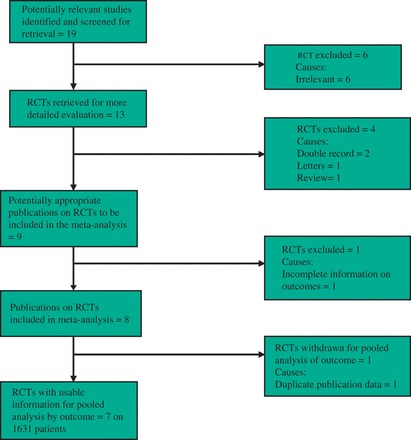
PRISMA flow chart showing trial selection methodology.

The PRISMA flow chart to explain the literature search strategy and trial selection is given in Figure 1. Seven randomized, controlled trials recruiting 1631 patients were retrieved from commonly used standard medical electronic databases [32–38]. There were 760 patients in the ABS group and 871 patients in the SS group. The characteristics of the included trials are given in Table 1. The salient features and treatment protocols adopted in the included randomized, controlled trials are given in Table 2. The short summary of data, selected primary and secondary outcome measures used to achieve a summated statistical effect are given in Table 3.

**Table 1:** Characteristics of included trials

Trial	Type of trial	Country	Surgical procedure	Comparison groups	Follow-up duration
Chatchai [32]	RCT	Thailand	Appendectomy	Triclosan-coated polyglactin 910	1 year
				vs	
				Traditional coated polyglactin 910	
Ford [33]	RCT	USA	All general surgical procedures	Triclosan-coated polyglactin 910	80 ± 5 days
				vs	
				Traditional coated polyglactin 910	
Galal [34]	RCT	Egypt	Across all surgical specialties	Triclosan-coated polyglactin 910	12 months
				vs	
				Conventional polyglactin 910	
Isik [35]	RCT	Turkey	Cardiothoracic	Triclosan-coated polyglactin 910	30 days
				vs	
				Traditional coated polyglactin 910	
Rašić [36]	RCT	Croatia	Open elective colorectal operations	Triclosan-coated polyglactin 910	Not recorded
				vs	
				Conventional polyglactin 910	
Williams [37]	RCT	UK	Breast surgery	Triclosan-coated polyglactin 910	6 weeks
			(Cancer)	vs	
				Conventional polyglactin 910	
Zhang [38]	RCT	China	Modified radical mastectomy	Triclosan-coated polyglactin 910	90 days ± 7
				vs	
				Chinese silk suture

**Table 2:** Treatment protocol adopted in included trials

Trial	AMS	Control
Chatchai [32]	Patients with appendicitis Tricosan-coated polyglactin 910 suture Prophylactic antibiotics given iv 30–60 mins prior to operation Study suture selected to close the abdominal sheath Appendectomy done with standard technique	Patients with appendicitis Polyglactin 910 suture Prophylactic antibiotics given IV 30–60 mins prior to operation Study suture selected to close the abdominal sheath Appendectomy done with standard technique
Ford [33]	Paediatric patients undergoing various general surgical procedures Triclosan-coated polyglactin 910 suture	Paediatric patients undergoing various general surgical procedures Traditional coated polyglactin 910 suture
Galal [34]	Patients selected from all surgical specialties Triclosan-coated polyglactin 910 suture	Patients selected from all surgical specialties Conventional polyglactin 910 suture
Isik [35]	Patients undergoing cardiac surgery Wound closure with antibacterial polyglactin 910 suture	Patients undergoing cardiac surgery Wound closure with traditional polyglactin 910 suture
Rasic [36]	Patients undergoing elective surgery for colorectal cancer Pre-op investigation included complete colonoscopy, CXR, CT and relevant serum tests Ops performed through a midline incision: skin incised with a scalpel; all other layers were transected with diathermy Prophylactic abxs given during induction of anaesthesia Wound closure was performed with a continuous single-layer mass technique (peritoneum, muscles and fascia) Triclosan-coated polyglactin 910 suture Skin closed with polyamide	Patients undergoing elective surgery for colorectal cancer Pre-op investigation included complete colonoscopy, CXR, CT and relevant serum tests Ops performed through a midline incision: skin incised with a scalpel; all other layers were transected with diathermy Prophylactic abxs given during induction of anaesthesia Wound closure was performed with a continuous single-layer mass technique (peritoneum, muscles and fascia) Polyglactin 910 suture Skin closed with polyamide
Williams [37]	Breast cancer surgery Subcutaneous triclosan-coated polyglactin 910 and poliglecaprone 25 Wounds dressed with Steri-Strips and Tegaderm or Cosmopore or Primapore	Breast cancer surgery Subcutaneous standard coated polyglactin 910 and poliglecaprone 25 Wounds dressed with Steri-Strips and Tegaderm or Cosmopore or Primapore
Zhang [38]	Patients undergoing modified radical mastectomy Intradermal closure Triclosan-coated polyglactin 910 suture	Patients undergoing modified radical mastectomy Simple interrupted closure Chinese silk suture

**Table 3:** Outcome variables

Variables	Chatchai 2009 [32]	Ford 2005 [33]	Galal 2011 [34]	Isik 2011 [35]	Rašić 2011 [36]	Williams 2011 [37]	Zhang 2011 [38]
**Patients (*n*)**							
**ABS**	50	98	230	170	91	74	47
**SS**	50	49	220	340	93	73	46
**Operation time (minutes)**		Not reported	Not reported	Not reported		Not reported	Not reported
**ABS**	41 ± 21.6				95.5 ± 17.3				
**SS**	45 ± 21.6				91.3 ± 18.6		
**SSI (*n*)**							
**ABS**	5	0	17	9	4	11	2
**SS**	4	3	33	22	12	9	5
**Length of stay (days)**		Not reported	Not reported	Not reported		Not reported	Not reported
**ABS**	3.7 ± 0				13.2 ± 1.3				
**SS**	3.7 ± 0				21.4 ± 2.8		
**Complications (*n*)**			Not reported	Not reported		Not reported	
**ABS**	0	17			1		15
**SS**	0	10			7		21

### Methodological quality of included studies

**Figure 2:**
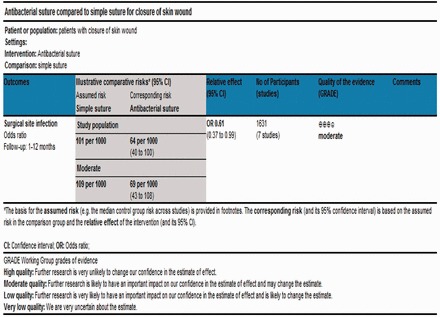
Strength and summary of the evidence analysed on GradePro®.

According to Jadad *et al.* and Chalmers *et al.* [29, 30], the quality of the majority of included trials was moderate due to the inadequate randomization technique, adequate allocation concealment, power calculations, blinding and intention-to-treat analysis [Table 4]. Based on the quality of included randomized, controlled trials, the strength and summary of evidence analysed on GradePro® [31] is given in Figure 2.

**Table 4:** Quality assessment of included trials

Trial	Randomization technique	Power calculations	Blinding	Intention-to-treat analysis	Concealment
Chatchai [32]	Random table	Yes	Yes	Not documented	Yes
Ford [33]	Computer generated	No	No	Not documented	No
Galal [34]	Computer generated, sealed pack for suture	No	Yes	Not documented	Yes
Isik [35]	Sequential? technique	Yes	Unable to determine	Not documented	Unable to determine
Rašić [36]	Computer generated, blind envelope system for suture	No	Yes	Not documented	Yes
Williams [37]	Computer generated, sequential envelope system for suture	Yes	Yes	Not documented	Yes
Zhang [38]	Computer generated	No	Yes	Yes	Yes

### Primary outcomes measures

#### Surgical site infection

**Figure 3:**
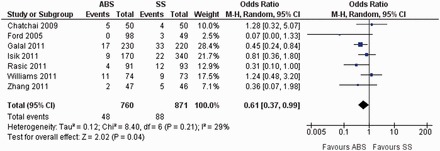
Forest plot for surgical site infection following the use of ABS versus SS. Risk ratios are shown with 95% confidence intervals. ABS = antibacterial suture; SS = simple suture.

Seven randomized, controlled trials contributed to the combined calculation of this variable [32–38]. There was minimal heterogeneity (Tau^2^ = 0.12, chi^2^ = 8.40, df = 6, [*P =* 0.21]; I^2^ = 29%) among trials. In the random-effects model (OR, 0.61; 95% CI, 0.37, 0.99; z = 2.02; *P <* 0.04; Figure 3), the risk of developing SSI following the use of ABS for skin wound closure was statistically lower compared to SS.

### Secondary outcomes measures

#### Postoperative complications

**Figure 4:**
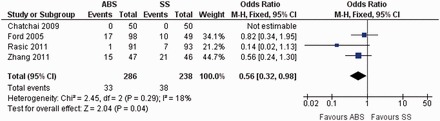
Forest plot for postoperative complications following the use of ABS versus SS. Risk ratios are shown with 95% confidence intervals. ABS = antibacterial suture; SS = simple suture.

All postoperative conditions (excluding SSI) leading to either delayed discharge of the patients or requiring medical or surgical intervention to treat—such as urinary tract infection, lower respiratory tract infection, cardiac or respiratory events and general anaesthesia-related complications—were jointly analysed as ‘postoperative complications’. Four randomized, controlled trials contributed to the combined calculation of this variable [32, 33, 36, 38]. There was minimal heterogeneity (Tau^2^ = 2.45, chi^2^ = 2.0, df = 2, [*P =* 0.29]; I^2^ = 18%) among trials. In the random-effects model (OR, 0.56; 95% CI, 0.32, 0.98; z = 2.04; *P <* 0.04; Figure 4), the risk of developing postoperative complications was statistically lower in the ABS group.

#### Duration of operation

**Figure 5:**
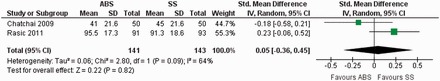
Forest plot for duration of operation following the use of ABS versus SS. Standardized mean differences are shown with 95% confidence intervals. ABS = antibacterial suture; SS = simple suture.

Two randomized, controlled trials contributed to the combined calculation of this variable [32, 36]. There was significant heterogeneity (Tau^2^ = 0.06; chi^2^ = 2.80, df = 1, [*P <* 0.09]; I^2^ = 64%) among trials. Therefore, in the random-effects model (SMD, 0.05; 95% CI, −0.36, 0.45; z = 0.22; *P =* 0.82; Figure 5), the duration of operation for both approaches was similar.

#### Length of hospital stay

**Figure 6:**
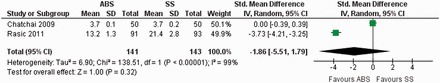
Forest plot for length of hospital stay following the use of ABS versus SS. Standardized mean differences are shown with 95% confidence intervals. ABS = antibacterial suture; SS = simple suture.

Two randomized, controlled trials contributed to the combined calculation of this variable [32, 36]. There was significant heterogeneity (Tau^2^ = 6.90; chi^2^ = 138.51, df = 1, [*P <* 0.00001]; I^2^ = 90%) among trials. Therefore, in the random-effects model (SMD, -1.85; 95% CI, -5.51, 1.79; z = 1.0; *P =* 0.32; Figure 6), the duration of hospital stay for both approaches was similar.

## DISCUSSION

This systematic review demonstrates that the use of ABS for skin closure in surgical patients is an effective measure in reducing the risk of postoperative surgical site infections and postoperative complications. ABS is comparable with SS in terms of length of hospital stay and duration of operation. Therefore, it may be used more judiciously to counteract the economic, cosmetic and morbidity-related issues arising from SSI.

There are several limitations to the present review. Randomized, controlled trials with fewer patients in this review may not have been sufficient to recognise small differences in outcomes. Quality of included trials was not good, due to inadequate randomization technique, allocation concealment, power calculations, blinding and intention-to-treat analysis [Table 4]. Variables like health-related quality of life measurement and cosmetic score should have been considered due to long-term psychological and social consequences of SSI. Studies evaluating cost-effectiveness should also be considered before recommending the routine use of ABS for skin closure in surgical patients. This analysis involved the trials run in various surgical specialties, which may be a source of bias. There was insufficient information regarding the use of various confounding interventions in both arms of included randomized, controlled trials, such as use of prophylactic antibiotics, timing and duration of antibiotics and, more importantly, the use of wound protectors. These confounding interventions can directly influence the incidence of SSI and may be a source of bias in the summated conclusion of this article, since a majority of the variables showed significant heterogeneity among included trials and trials are very diverse in terms of inclusion criteria, exclusion criteria, clinical and methodological patterns. The majority of the variables showed significant heterogeneity among included trials because they are very diverse in terms of inclusion criteria, exclusion criteria and in clinical as well as methodological patterns. While there are statistically significant findings using the random-effects model, with a lower rate of SSI associated with the use of ABS, the clinical significance and cost–benefit significance remains unknown. Causes of reduced SSI in ABS are apparent due to the presence of antibiotics at wound sites preventing microbial colonization. However, it is difficult to explain the similar length of stay in both groups despite the reduced incidence of SSI in the ABS group. There may be many reasons behind this outcome. In the majority of cases, diagnosis of SSI is made in the community and therefore it would not influence the length of stay. Variable follow-up, the diverse group of patients analysed summatively in this review and statistically significant heterogeneity among trials in case of length stay may all be responsible for this difference. The development of SSI is multifactorial, making it extremely difficult to account for the different confounding factors and reducing bias even in a well designed, randomized, controlled trial. This task becomes significantly more challenging when a systematic review of highly heterogeneous studies—like our meta-analysis—is undertaken. The aetiopathogenesis of SSI can be influenced by i) patient-dependent factors such as immunosuppression, hypoalbuminemia, use of steroids, diabetes mellitus, renal failure, hepatic failure, and congestive cardiac failure, ii) surgeon-related factors including proper sterility, hand washing, surgical technique and iii) type of operation, such as clean, clean contaminated, contaminated and dirty. These factors are difficult to randomize and a study such as this, that reviews various surgical techniques, specialties and patient population, may be of little help.

Although our conclusion is based on the summated outcome of seven randomized, controlled trials, it should be considered cautiously because the quality of the majority of included trials was poor. There is still a lack of stronger evidence to support the routine use of ABS but it can be considered an alternative and may initially be applied in selected groups of patients. A major, multicentre, randomized, controlled trial of high quality according to CONSORT guidelines is mandatory to validate these findings.

**Conflict of interest:** none declared.

## References

[1] Walter CJ, Dumville JC, Sharp CA (2012). Systematic review and meta-analysis of wound dressings in the prevention of surgical-site infections in surgical wounds healing by primary intention. Br J Surg.

[2] Health Protection Agency Surveillance of surgical site infection in English hospitals 1997–2002. A national surveillance and quality improvement programme.

[3] Wick EC, Vogel JD, Church JM (2009). Surgical site infections in a ‘high outlier’ institution: are colorectal surgeons to blame?. Dis Colon Rectum.

[4] Boltz MM, Hollenbeak CS, Julian KG (2011). Hospital costs associated with surgical site infections in general and vascular patients. Surgery.

[5] Berard F, Gandon J (1964). Postoperative wound infections: the influence of ultraviolet irradiation of the operating room and of various other factors. Ann Surg.

[6] McLaws ML, Murphy C, Whitby M (2000). Standardising surveillance of nosocomial infections: the HISS program. Hospital Infection Standardised Surveillance. J Qual Clin Pract.

[7] National Institute for Health and Clinical Excellence (NICE) Surgical Site Infection: Prevention and Treatment of Surgical Site Infection.

[8] Horan TC, Andrus M, Dudeck MA (2008). DC/HNSN surveillance definition of healthcare-associated infection and criteria for specific types of infection in the acute care setting. Am J Infect Control.

[9] Poandl TM A 21-day exploratory pilot study comparing the cosmetic result of subcutuicular MONOCRYL Plus* versus transcutaneous Chinese silk for incisional wound closure in a pig model.

[10] Nandi PL, Soundara Rajan S, Mak KC (1999). Surgical wound infection. Hong Kong Med J.

[11] Dancer SJ, Stewart M, Coulombe C (2012). Surgical site infections linked to contaminated surgical instruments. J Hosp Infect.

[12] Lin GL, Qiu HZ, Xiao Y (2012). Safety and efficacy of prophylactic single antibiotics administration in selective open colorectal surgery. Zhonghua Wei Chang Wai Ke Za Zhi.

[13] Cannon JA, Altom LK, Deierhoi RJ (2012). Preoperative oral antibiotics reduce surgical site infection following elective colorectal resections. Dis Colon Rectum.

[14] Junker T, Mujagic E, Hoffmann H (2012). Prevention and control of surgical site infections: review of the Basel Cohort Study. Swiss Med Wkly.

[15] Phatak UR, Pedroza C, Millas SG (2012). Revisiting the effectiveness of interventions to decrease surgical site infections in colorectal surgery: A Bayesian perspective. Surgery.

[16] Russell AD (2004). Whither triclosan?. J Antimicrob Chemother.

[17] McMurry LM, Levy SB (1998). Triclosan targets lipid synthesis. Nature.

[18] Levy CW, Roujeinikova A, Sedelnikova S (1999). Molecular basis of triclosan activity. Nature.

[19] Marco F, Vallez R, Gonzalez P (2007). Study of the efficacy of coated Vicryl plus antibacterial suture in an animal model of orthopedic surgery. Surg Infect (Larchmt).

[20] Storch ML, Rothenburger SJ, Jacinto G (2004). Experimental efficacy study of coated VICRYL plus antibacterial suture in guinea pigs challenged with Staphylococcus aureus. Surg Infect (Larchmt).

[21] Rothenburger S, Spangler D, Bhende S (2002). In vitro antimicrobial evaluation of Coated VICRYL* Plus Antibacterial Suture (coated polyglactin 910 with triclosan) using zone of inhibition assays. Surg Infect (Larchmt).

[22] http://www.cochrane-handbook.org.

[24] DerSimonian R, Laird N (1986). Meta-analysis in clinical trials. Control Clin Trials.

[25] DeMets DL (1987). Methods for combining randomized clinical trials: strengths and limitations. Stat Med.

[26] Higgins JP, Thompson SG (2002). Quantifying heterogeneity in a meta-analysis. Stat Med.

[27] Egger M, Smith GD, Altman DG Systematic Reviews in Healthcare.

[29] Jadad AR, Moore RA, Carroll D (1996). Assessing the quality of reports of randomized clinical trials: is blinding necessary?. Control Clin Trials.

[30] Chalmers TC, Smith H, Blackburn B (1981). A method for assessing the quality of a randomized control trial. Control Clin Trials.

[31] http://ims.cochrane.org/revman/otherresources/.

[32] Chatcahi M, Ungbhakorn P, Paocharoen V (2009). Efficacy of antimicrobial coating suture coated polyglactin 910 with triclosan (Vicryl Plus) compared with polyglactin in reduced surgical site infection of appendicitis, double blind randomized control trial, apreliminary safety report. J Med Assoc Thai.

[33] Ford HR, Jones P, Gaines B (2005). Intraoperative handling and wound healing: controlled clinical trial comparing coated VICRYL plus antibacterial suture (coated polyglactin 910 suture with triclosan) with coated VICRYL suture (coated polyglactin 910 suture. Surg Infect (Larchmt).

[34] Galal I, El-Hindawy K (2011). Impact of using triclosan-antibacterial sutures on incidence of surgical site infection. Am J Surg.

[35] Isik I, Selimen D, Senay S (2012). Efficiency of antibacterial suture material in cardiac surgery: a double-blind randomized prospective study. Heart Surg Forum.

[36] Rasić Z, Schwarz D, Adam VN (2011). Efficacy of antimicrobial triclosan-coated polyglactin 910 (Vicryl* Plus) suture for closure of the abdominal wall after colorectal surgery. Coll Antropol.

[37] Williams N, Sweetland H, Goyal S (2011). Randomized trial of antimicrobial-coated sutures to prevent surgical site infection after breast cancer surgery. Surg Infect (Larchmt).

[38] Zhang ZT, Zhang HW, Fang XD (2011). Cosmetic outcome and surgical site infection rates of antibacterial absorbable (polyglactin 910) suture compared to Chinese silk suture in breast cancer surgery: a randomized pilot research. Chin Med J (Engl).

